# Perceptions and Attitudes of Chinese Oncologists Toward Endorsing AI-Driven Chatbots for Health Information Seeking Among Patients with Cancer: Phenomenological Qualitative Study

**DOI:** 10.2196/71418

**Published:** 2025-07-23

**Authors:** Lijuan Zeng, Qiaoqi Li, Yan Zuo, Ying Zhang, Zhaojun Li

**Affiliations:** 1 Division of Abdominal Tumor Multimodality Treatment, Cancer Center, West China Hospital, Sichuan University, Chengdu, China; 2 Department of Gynecology and Obstetrics Nursing, West China Second University Hospital, Sichuan University, Chengdu, China; 3 West China School of Nursing, Sichuan University, Chengdu, China; 4 Key Laboratory of Birth Defects and Related Disease of Women and Children, Sichuan University, Chengdu, China; 5 Division of Internal Medicine, Institute of Integrated Traditional Chinese and Western Medicine, West China Hospital, Sichuan University, Chengdu, China; 6 Department of Radiation Oncology, Hainan Affiliated Hospital of Hainan Medical University (Hainan General Hospital), #19 Xiuhua Road, Xiuying District, Haikou, 570311, China, 86 18898963083

**Keywords:** artificial intelligence, attitude, chatbot, health information seeking, large language model, liability, misinformation, oncologist, perception, patient education, qualitative study

## Abstract

**Background:**

Chatbots driven by large language model artificial intelligence (AI) have emerged as potential tools to enhance health information access for patients with cancer. However, their integration into patient education raises concerns among oncologists. Limited literature has examined the perceptions and attitudes of oncologists in terms of endorsing AI-driven chatbots for health information.

**Objective:**

This study aims to explore the perceptions and attitudes of Chinese oncologists toward endorsing AI-driven chatbots to patients with cancer.

**Methods:**

In this phenomenological qualitative study, we purposively sampled oncologists from 4 hospitals in Southwest and East China and conducted semistructured interviews with 24 participants between November 19, 2024, and December 21, 2024. The data saturation principle was observed to determine the end point of data collection. Data were analyzed using the Colaizzi method.

**Results:**

The participants were aged 42.0 (range 29‐53) years on average, including 9 (37%) female and 15 (62%) male participants. The participants had an average of 8.8 (range 1‐25) years in oncology. Of the participants, 7 (29%) had recommended AI chatbots to patients. Three key themes were revealed from analysis of interview transcriptions, including perceived benefits, significant concerns, and impacts on doctor-patient dynamics. Benefits included enhanced accessibility and potential support for chronic condition management. Concerns centered on liability, misinformation, lack of personalization, privacy and data security risks, and patient readiness and education. Oncologists stressed a dual impact of AI chatbots on doctor-patient dynamics, recognizing the potential for improved communication and risks of trust erosion due to overreliance on AI.

**Conclusions:**

While recognizing the potential of AI-driven chatbots to enhance accessibility of health information and chronic disease management, Chinese oncologists report significant concerns, including liability, misinformation, lack of personalization, privacy and data security risks, and patient readiness. Addressing the challenges requires comprehensive solutions, such as clear policies and guidelines, rigorous testing and validation, institutional endorsement, and robust patient and provider education. Future efforts should focus on resolving the barriers while leveraging the strengths of AI technology to support patient-centered care in a safe, effective, and ethical manner.

## Introduction

Chatbots driven by large language model (LLM) artificial intelligence (AI) have emerged as innovative tools in health care [1-3]. They leverage advanced natural language processing to interact with users in real-time. They simulate human conversation to provide information, answer queries, and offer support based on a vast repository of data [4]. Equipped with LLMs, they are capable of understanding complex questions and delivering contextually relevant responses. This makes them valuable assets for health information dissemination [3].

The integration of AI-driven chatbots in health care has been growing rapidly, given their potential to bridge gaps in patient education and support. The tools can assist individuals in understanding medical conditions, exploring treatment options, and navigating health systems. Their ability to operate 24/7 offers a unique advantage in addressing patients’ nonurgent concerns and reducing reliance on in-person consultations for routine information [5-7]. Additionally, they hold promise in alleviating the burden on health care providers by streamlining communication and enhancing patient engagement [8,9]. Globally, widely recognized AI-driven chatbots, such as ChatGPT, have gained prominence for their versatility in responding to health-related inquiries [10]. In China, domestic tools such as Kimichat have been developed to cater to local linguistic and cultural contexts.

Accessible and reliable health information is crucial for patients with cancer. It enables them to make informed decisions about their treatment, manage symptoms, and improve their overall quality of life. In cancer care, patients often face complex medical decisions that require a clear understanding of their condition, treatment options, potential side effects, and prognosis [11,12]. Reliable information can also help patients and their families cope with the emotional and psychological challenges associated with a cancer diagnosis and foster a sense of control and preparedness [13,14]. However, patients with cancer frequently encounter significant challenges in accessing relevant information. One major issue is the prevalence of misinformation, particularly from unverified web-based sources. Patients who turn to search engines or social media platforms for answers may encounter inaccurate, incomplete, or overly generalized information that can mislead them or exacerbate their anxiety [15,16]. Additionally, limited access to health care resources further complicates information-seeking efforts. Oftentimes, face-to-face consultations with oncology specialists may be infrequent or unavailable. Patients may struggle to attain timely answers, which leaves gaps in their understanding of their disease and its management. Even in urban centers, the high patient-to-doctor ratio often results in brief consultations, leaving little time for detailed explanations of medical conditions or treatments. As a result, innovative solutions, such as AI-driven chatbots, can provide reliable and easily accessible health information to support patients with cancer, which could complement traditional health care delivery.

According to current reports, LLM AI chatbots, such as ChatGPT, have significantly impacted patient education by providing accessible health information and personalized support. They have been used to interpret complex medical data, generate patient-friendly educational materials, and answer health-related queries, which has enhanced patient engagement and health literacy [17]. However, evaluations of the chatbots reveal mixed results regarding accuracy and reliability. For instance, studies assessing ChatGPT’s responses to medical queries have noted variability in accuracy, safety, relevance, and readability. Challenges such as the risk of disseminating misinformation, lack of personalization, and ethical concerns related to patient privacy have been noted [18-20].

Oncologists are at the forefront of cancer care. They serve as clinical decision makers and trusted advisors in patient education. Their role has evolved to include guiding patients through increasingly complex treatment options and emerging digital health tools. In today’s rapidly changing health care landscape, the endorsement of innovative technologies, such as AI-driven chatbots, is critical, as it can significantly influence patient trust and engagement. Despite their central impact on patient care and information dissemination, the unique perspectives of oncologists on these digital tools have received limited attention. This study seeks to bridge that gap by exploring Chinese oncologists’ perceptions and attitudes toward endorsing AI-driven chatbots for the health information seeking of patients with cancer. By using a phenomenological qualitative approach, our findings may capture their lived experiences, illuminate the pivotal role they play in integrating digital innovations into clinical practice, and identify both the facilitators and barriers to adoption.

## Methods

### Study Setting

The study was conducted among oncologists working in 4 major hospitals in Southwest and East China. The primary investigator (PI; first author) is from West China Hospital, which is one of the country’s largest comprehensive hospitals and a leading regional center for cancer care. The other 3 hospitals included 2 major comprehensive hospitals and one specialized cancer hospital, all recognized for their strong oncology services. The hospitals were chosen to ensure diverse representation of oncologists’ experiences and perspectives, given a wide range of institutional settings and patient populations.

### Study Design

We used a phenomenological qualitative design to explore the perceptions and attitudes of oncologists toward endorsing AI-driven chatbots to their patients for health information seeking. The phenomenological approach was chosen for its ability to capture and interpret the subjective experiences and insights of participants and provide a deeper understanding of the study topic [21]. The design was deemed appropriate for investigating the attitudes and concerns of oncologists in the context of integrating AI technologies into patient education and care.

### Sampling Strategy

A purposive sampling strategy was used, which ensured the selection of participants who had appropriate backgrounds and could provide relevant, rich, and detailed insights [22].

### Participant Enrollment

Licensed oncologists actively practicing at the selected hospitals, with at least 2 years of clinical experience, were recruited for the study. Participants were required to have experience in providing patient education and using AI-driven chatbots, including ChatGPT or similar Chinese chatbots. Those who expressed interest in participating, after being given detailed study information, were enrolled.

Notably, we set a minimum requirement of 2 years of clinical experience to ensure that participants had adequate exposure to the complexities of real-world clinical practice, as well as exposure to the AI chatbots emerging in the last 2 years. The threshold was meant to ensure that the collected data are grounded in experienced perspectives, thereby enhancing the credibility and validity of the findings by relying on the judgments of practitioners who have encountered realistic clinical scenarios and patient interactions.

To recruit participants, invitations were extended via phone calls through the PI’s professional network. Given the PI’s senior standing in oncology and long-standing relationships with a wide range of practitioners across different hospital settings in Southwest and East China, the purposive sampling strategy enabled the inclusion of oncologists with diverse demographic backgrounds, clinical experiences, and levels of exposure to AI-driven chatbots. This targeted approach ensured a comprehensive representation of perspectives, thereby mitigating potential recruitment bias related to network-based sampling. A 20‐30 minute semistructured interview was conducted either immediately or at a later time convenient for the participant.

### Data Collection

Data were collected through semistructured phone interviews. An interview guide was developed and pilot-tested with 3 oncologists to refine the questions and flow (Multimedia Appendix 1). The pilot interviews and transcripts were excluded from the final analysis. Each interview phone call was audio-recorded with permission. Notes were also taken to capture key observations. Data collection continued until data saturation was achieved, as determined by the point where no new analytic information emerged in 3 consecutive interviews [23]. Participants’ demographic data were collected before interviews using a standard demographic information form, which was compiled and desensitized for subsequent analysis.

### Data Analysis

Data were analyzed using the Colaizzi method, a rigorous approach that systematically guides researchers through extracting and organizing significant statements, formulating meanings, and clustering themes [24]. We chose the Colaizzi method over other phenomenological techniques, such as Giorgi or van Manen approaches, for several reasons. First, unlike the other 2 methods, the practice of returning to participants for validation of the identified meanings enhances the credibility and confirmability of our findings. This was especially important as our findings might be used to support sensitive clinical decisions. Second, the Colaizzi method is particularly suited for capturing the in-depth details of lived experiences. This aligns with our objective of exploring the complex perceptions and attitudes of oncologists. It could ensure a comprehensive interpretation of the data while maintaining comprehensive transparency throughout the analysis, which strengthens the study’s methodological rigor. The process involved multiple steps. The researchers first read the transcripts at least 3 times to gain familiarity and then extracted significant statements and formulated meanings. The meanings were clustered into themes and subthemes, which were returned to participants for validation. Any discrepancies were discussed until consensus was achieved.

### Study Rigor

To ensure the trustworthiness of the study, we implemented several strategies, including member checking for ensuring credibility, where participants reviewed and validated the findings; an audit trail documenting key decisions in the study process for dependability; regular reflexivity discussions among the research team to acknowledge and mitigate potential biases due to their personal and professional backgrounds and assumptions; and a reflexivity statement for outlining the researchers’ positions, potential influences on the study, and steps taken to minimize bias throughout the research process (Multimedia Appendix 2) [25-27]. Although we did not formally assess intercoder reliability or conduct external validation of the coding process, multiple researchers independently coded the data and subsequently discussed discrepancies until consensus was reached.

### Ethical Considerations

The study was ethically approved by the Ethics Committee of West China Hospital, Sichuan University (HXLL0751). Verbal informed consent was obtained from each participant at the beginning of their interview, including explicit permission for the interview to be recorded. The consent process was documented as part of the audio recording. The study was conducted in accordance with the principles of the Declaration of Helsinki and relevant regulatory codes and guidelines for human subject protection. No compensation of any form was provided to participants for their participation.

All identifiers were anonymized. Participants were assigned unique identifiers (eg, P1 and P2). Audio recordings and transcripts were stored securely on a password-protected flash drive, which was kept by the PI and was accessible only to the research team. Consent forms and other sensitive documents were stored in a locked drawer in the PI’s office. Participants were informed about their right to withdraw from the study at any time without any repercussions.

## Results

### Overview

A total of 29 candidate oncologists were contacted, with 5 (17%) of them declining participation due to unavailability for interview. Eventually, 24 oncologists were interviewed between November 19, 2024, and December 21, 2024, by which time data saturation was achieved. The interviews lasted 21.7 (range 16‐25) minutes on average. Notably, although the interviews were relatively short given the phenomenological nature of this study, the depth and richness of the data were not compromised as the busy schedules and direct communication style of Chinese oncologists enabled them to convey focused, meaningful insights efficiently. The participants were aged 42.0 (range 29‐53) years on average, including 9 (37%) female and 15 male (62%) participants. The participants had an average of 8.8 (range 1‐25) years in oncology. Of the participants, 7 (29%) had recommended AI chatbots to patients. Table 1 summarizes the demographic characteristics of participants.

**Table 1.** Summary of participant demographic characteristics.

Characteristics		Value (N=24), n (%)	
Sex			
Female		9 (37)	
Male		15 (62)	
Age (range in years)			
<35		6 (25)	
35‐45		9 (37)	
>45		9 (37)	
Prior exposure to AI^a^ chatbots			
Chinese chatbots		7 (29)	
ChatGPT and Chinese chatbots		17 (71)	
Ever recommended an AI chatbot to a patient			
Yes		7 (29)	
No		17 (71)	
Expressed reluctance to use an AI chatbot in clinical practice			
Yes		19 (79)	
No		5 (21)	

a AI: artificial intelligence.

### Key Themes

Three overarching themes with 7 subthemes emerged from the interviews, including the perceived benefits of AI chatbots, 5 significant concerns such as liability, misinformation, lack of personalization, patient privacy, and patient readiness, and the impact of chatbots on the doctor-patient relationship.

#### Theme 1: Perceived Benefits of AI Chatbots

Participants cited 2 main potential benefits of AI chatbots in oncology care regarding their ability to enhance accessibility and support chronic disease management. While most participants saw them generally as supplementary to professional care, some recognized their value in improving patient education and engagement, especially for routine queries or long-term disease management.

##### Subtheme 1.1: Accessibility and Convenience

The 24/7 availability of AI chatbots was the most frequently mentioned advantage. Participants noted that the tools could help bridge gaps in access to health information. By addressing nonurgent queries, they were believed to be an effective asset to reduce the workload of health care providers while enabling patients to seek basic information independently.

*One biggest advantage [of the AI chatbots] is that they are readily available. Patients can access them at any time, even late at night. For minor concerns, such as looking up symptoms or side effects, patients don’t have to wait to see a doctor*.

[P2]

*It’s not feasible for patients to call a hospital or doctor for small queries. The AI [chatbots] is particularly useful in this situation. Patients can get immediate answers to their questions*.

[P7]

##### Subtheme 1.2: Potential for Chronic Condition Management

Participants also recognized the potential for AI chatbots to support patients managing chronic conditions, as a result of their high availability. By providing timely answers when patients have concerns or questions in their chronic disease management, the tools could help them adhere to treatment plans more effectively.

*Patients with cancer have many questions about their care and rehabilitation on a daily basis. If the questions are not answered timely, they might lose track of how to manage their care effectively. The AI [chatbots] can fill this gap. They can provide instant answers, for example about new symptoms, medications, side effects, or even dietary recommendations*.

[P10]

### Theme 2: Concerns

Participants expressed significant concerns about integrating AI chatbots into real-world oncology care. The concerns primarily revolved around issues of liability, accuracy, lack of personalization, patient privacy and data security, and the readiness of patients to use the tools effectively. Some participants even emphasized that the challenges must be addressed before chatbots can be widely recommended or trusted in clinical practice.

#### Subtheme 2.1: Liability Issues

The question of accountability was a recurring concern among most participants, even those who had already recommended the tools to patients. Oncologists were uncertain about who would bear responsibility if patients experienced adverse outcomes after following chatbot recommendations. This led to hesitation in endorsing or recommending them in clinical practice.

*For certain, liability is the biggest concern. If a patient follows advice given by AI and something goes wrong, who will be held responsible? As a doctor, I cannot recommend a tool to patients unless I myself am certain whether it’s safe and reliable and know that I’m safe from the liabilities. Too much risk at this point*.

[P6]

*Patients are not [medical] professionals. They are likely to assume that the information from a tool recommended by a doctor or hospital is trustworthy. But what if the AI [chatbot] provides wrong advice? such as incorrect source material or a programming error? It’s not just the chatbot developers who will face troubles. We doctors, too. What if something goes wrong with the patient after they follow the AI [chatbot’s] advice, for example, taking a wrong medication? Who will be held responsible?*

[P19]

#### Subtheme 2.2: Misinformation Risk

Another frequently raised concern was the risk of chatbots providing inaccurate or misleading information. This was particularly worrisome for patients with low health literacy, who might misinterpret chatbot responses or take them at face value without consulting a health care professional.

*I have always been skeptical about the AI [chatbot’s] responses. Can they be such as outdated or incorrect because of wrong source data*.

[P11]

*Sometimes, AI [chatbots] can oversimplify complex medical information. For example, they might give a generic explanation of symptoms that doesn’t account for the patient’s conditions. It’s fine if the patient doesn’t act on it, but what if the patient doesn’t really understand it and becomes unwilling to see a doctor because they already have the “answer.”*

[P24]

#### Subtheme 2.3: Lack of Personalization

Several participants indicated concern about the inability of chatbots to provide personalized recommendations. They emphasized that oncology care often requires advice tailored to individual patient needs that cannot be generalized.

*Every patient is unique, especially in oncology. I have tried the AI [chatbots] myself. Most of them don’t functionalities to enter information on patient’s personal conditions. The responses are generic... Yes, you can manually enter a patient’s condition, but it takes a lot of work and you have to enter it every time in a new chat session*.

[P5]

#### Subtheme 2.4: Patient Privacy and Data Security

Patient privacy and data security concerns were another significant issue raised by participants. Oncologists were wary about how patient data would be collected, stored, and used by AI systems and the companies behind them, especially given the sensitive nature of medical data.

*The state is very serious about protecting patient privacy and medical data security. We are completely blind when it comes to the AI [chatbots]. Even if the patient is willing to enter their medical data, how do you know what the AI or the company would do with it? What if the information is leaked? Will I be held responsible because I recommended it to the patient?*

[P5]

#### Subtheme 2.5: Patient Readiness and Education

Participants emphasized that not all patients are equipped to use AI chatbots effectively. Factors such as age, technological literacy, and familiarity with digital tools could limit the accessibility of the technologies for certain populations.

*Some older patients don’t even know how to use their smartphones. It could be even more difficult for them to AI*.

[P7]

*There is this possible usability issue. Some patients, for example older patients or those who are not familiar with digital products, they may not be able to use the AI [chatbots] effectively*.

[P22]

### Theme 3: Impact on Doctor-Patient Dynamics

Participants discussed how chatbots could influence the dynamics of doctor-patient interactions. While some believed chatbots might enhance communication by helping patients prepare better for consultations.

*AI [chatbots] can help patients better prepare for subsequent consultations. For example, they may already have asked basic questions and gotten answers. This allows them to focus on more complex issues during the consultation*.

[P12]

On the other hand, some others expressed concern that overreliance on the tools could lead to conflicts or misunderstandings.

*There is a risk. Some patients might trust the chatbot more than the doctor. If the AI’s advice contradicts the doctor’s, this could create unnecessary tension, even undermine trust in us doctors*.

[P16]

## Discussion

### Principal Findings

In this qualitative study of Chinese oncologists, we investigated their perceptions and attitudes toward endorsing AI-driven chatbots to patients with cancer. Three key themes were revealed from analysis of interview transcriptions, including perceived benefits, significant concerns, and impacts on doctor-patient dynamics. Benefits included enhanced accessibility and potential support for chronic condition management. Concerns centered on liability, misinformation, lack of personalization, privacy and data security risks, and patient readiness and education. Oncologists stressed a dual impact of AI chatbots on doctor-patient dynamics, recognizing potential for improved communication and risks of trust erosion due to overreliance on AI.

### Comparison to Prior Work

According to our findings, fewer than a third of participants had recommended the tools to patients, despite all having used chatbots themselves. This hesitation reflects a cautious approach among oncologists, shaped by a balance between recognizing the potential benefits of chatbots, such as enhancing accessibility and supporting chronic disease management, and grappling with significant concerns, including liability, misinformation, lack of personalization, and privacy and data security concerns. The finding is consistent with prior reports on Chinese oncologists’ perceptions and attitudes [28,29]. There seems to be a gap between personal experience with AI tools and professional endorsement. This suggests significant challenges in integrating such technologies into real-world oncology care.

In addition to their high availability, a widely recognized strength of LLM AIs in current literature [30], participants emphasized the potential of AI chatbots to support chronic disease management, particularly in cancer care. Their 24/7 accessibility enables patients to address concerns promptly, which is essential for maintaining adherence to complex treatment plans. Participants noted that timely responses from chatbots could prevent patients from feeling neglected or uncertain. This may enhance their ability to manage their care independently. The finding is consistent with existing studies that LLM AIs improve aspects of chronic disease management [31-34]. AI chatbots are therefore promising as supplementary tools in oncology care, with the potential to improve patient engagement, treatment adherence, and overall outcomes.

As the main findings of this study, participants expressed substantial concerns about recommending AI chatbots to patients in real-world practice, including liability, misinformation, lack of personalization, patient privacy and data security, and the readiness of patients. The issue of liability was particularly prominent. In particular, many participants questioned who would be held accountable if patients experience adverse outcomes after following chatbot advice. Even those who had previously recommended chatbots were hesitant, citing liability concerns as a major barrier. Liability concerns are a common challenge when integrating new technologies into formal or professional processes.

In health care, previous studies have documented similar apprehensions with the adoption of telemedicine platforms and wearable health devices. For instance, in telemedicine implementation, questions arose regarding who would be held responsible if a remote consultation resulted in a misdiagnosis due to technological limitations [35]. Similarly, the use of wearable health monitors, such as fitness trackers or medical-grade devices such as continuous glucose monitors, has raised concerns about the accuracy of the data and the potential consequences of acting on incorrect or incomplete information [36]. Therefore, liability concerns are not unique to AI chatbots but are a recurring theme when technology intersects with professional accountability.

To address this issue, solutions at multiple levels are necessary. At the policy level, clear regulations and guidelines must be established to define the appropriate use of AI chatbots in clinical practice and delineate accountability in cases of adverse outcomes. Such policies should include standards for chatbot development, data validation, and evidence-based responses to ensure their reliability. Additionally, the involvement of professional regulatory bodies to formally approve and monitor the use of these tools can provide an added layer of accountability and confidence for both patients and providers.

At the institutional level, hospitals and health care organizations must play a pivotal role in facilitating the safe integration of AI chatbots. Formal endorsement by institutions, including selecting validated tools, development of hospital-endorsed chatbot guidelines, provision of AI literacy training for care providers and patients, and integrating them into existing clinical workflows, can help establish trust. Additionally, providing training for health care providers on how to endorse and monitor chatbot use and ensuring that patients are aware of the chatbot’s capabilities and limitations are essential steps. Such institutional involvement would shift some of the accountability burden from individual practitioners to a broader, system-level responsibility, thereby reducing hesitancy among health care providers.

Misinformation is a frequently reported concern with LLM AI chatbots, including issues related to accuracy, the quality of training datasets, the reliability of the source data, and so-called “hallucinations” [37]. LLMs might rely on vast and unfiltered internet data to generate responses, which can contain inaccuracies, biases, and outdated information [38,39]. Hallucinations are where the chatbot generates plausible-sounding but factually incorrect or irrelevant responses [40]. Additionally, the inherent variance in responses to similar prompts could further undermine their reliability [41,42]. These are realistic risks for patients seeking consistent and accurate information.

To mitigate the concerns, it is critical to test and validate AI chatbots rigorously before recommending them to patients. Health care institutions should select chatbots based on performance in controlled validation studies, focusing on accuracy, consistency, and their ability to provide evidence-based responses. If existing LLMs fail to meet clinical needs, institutions could consider customizing specialized LLM agents trained on validated medical datasets and updated guidelines specific to oncology. Regular updates and testing are also essential to ensure that chatbots remain aligned with current medical knowledge and best practices.

User feedback is another valuable tool for addressing misinformation. Implementing processes to gather and analyze feedback from both patients and health care providers can help identify and rectify inaccuracies. Improving the prompting skills can also enhance the accuracy of chatbot responses [43]. Training users to frame their queries effectively, such as including relevant context and specific details, can reduce ambiguities and improve the relevance of the chatbot’s answers. Providing tutorial materials or conducting workshops on effective questioning techniques would be a practical way to enhance user interactions with chatbots. Similarly, physicians should be equipped to guide patients on how to use chatbots responsibly and effectively. Finally, clear instructions on the limitations of AI chatbots should accompany their implementation. Patients must understand that chatbots are supplementary tools, not substitutes for professional medical advice.

The concern over the lack of personalization in AI chatbots may not be as substantial as perceived by participants. Improved prompting skills, where users provide specific details about their health conditions or concerns, can significantly enhance the relevance and accuracy of responses. For example, entering contextual information such as medications, symptoms, or treatment history can allow chatbots to tailor recommendations more effectively. Training both patients and health care providers to use precise and structured prompts can help bridge the gap in personalization.

Moreover, some chatbots are already equipped with functionalities to integrate personal health data to deliver more customized responses. For instance, iFlyhealth, a Chinese AI chatbot that allows users to input their medical records, health checkup reports, and other personal information, demonstrates the potential to provide contextually relevant guidance [44]. The functionalities enable chatbots to adapt their advice to the unique needs of individual users, especially in oncology care, where personalized care is critical.

However, the integration of personal health data introduces a parallel concern: patient privacy and data security. Participants in this study expressed concerns about the risk of sensitive health information being mishandled or accessed without consent. For chatbots to achieve meaningful personalization without compromising privacy, robust data security measures must be in place, such as end-to-end encryption, secure storage systems, and strict access controls. Additionally, transparent communication with users about how their data will be collected, stored, and used is essential to build trust and mitigate privacy concerns [45-47]. Balancing the benefits of personalization with the need for stringent privacy protections is a critical challenge for the adoption of AI chatbots in oncology care. While personalization enhances the utility, addressing privacy concerns is pivotal in ensuring their acceptance and widespread use. Future research should explore ways to achieve this balance, including the development of secure, locally hosted AI models with minimized data exposure.

Patient readiness and education are important considerations when adopting novel technologies such as AI chatbots [48,49]. However, this should not be overstressed to the detriment of broader implementation. While some patients, particularly older adults or those less familiar with technology, may face challenges in using chatbots, the principle of adoption should focus on serving the majority. Most patients are likely to adapt quickly. Those who are less ready should not be a cause to hinder the rollout of the tools. Instead, targeted efforts, such as simplified chatbot designs, caregiver assistance, or personalized training, can address their needs.

The integration of AI-driven chatbots in oncology care presents a dual impact on doctor-patient dynamics. On one hand, the tools offer enhanced accessibility and convenience, potentially improving patient preparation for consultations by addressing routine inquiries and freeing up time for more complex discussions. This can streamline communication and enhance the efficiency of health care delivery. On the other hand, there are concerns that patients might overrely on chatbots, potentially leading to trust issues if their advice contradicts that of health care providers. In the Chinese context, cultural factors may further influence how patients perceive and interact with the technologies. This finding aligns with prior reports [50-52]. Clear communication about the role and limitations of chatbots is essential, in addition to developing guidelines to ensure that such tools complement rather than replace human interaction. A balanced approach is crucial to harness the benefits of AI while preserving the integrity of the doctor-patient relationship in oncology care.

### Limitations and Future Directions

This study has several limitations. First, conducting semistructured interviews via phone calls inherently limits the collection of nonverbal cues, which may hinder a full understanding of the emotional context behind responses. Although the interviewer initiated a brief ice-breaking conversation to foster rapport, the absence of visual feedback remains a constraint. Second, the requirement of a minimum of 2 years of clinical experience ensured that participants had sufficient professional and technical exposure. However, it may have excluded newer practitioners who could offer innovative perspectives on the integration of digital health technologies. Future research should consider broadening the participant criteria to enhance representativeness. Third, we did not assess intercoder reliability or externally validate the coding process, but used independent coding and consensus among researchers. A more stringent process may further enhance the rigor of future studies. Fourth, as a qualitative study focused on a specific group of Chinese oncologists from selected hospitals, the findings should be interpreted and adopted with caution in other clinical settings or cultural contexts. For example, the dynamics of patient challenges to an oncologist’s judgment or issues of physician liability in endorsing an AI chatbot may differ across regions. Finally, as participants were known professionally to the interviewer, this familiarity might have inhibited candid discussion of controversial opinions and confined recruitment to those willing to engage in interviews, thereby narrowing the range of perspectives captured. These limitations should be taken into account when interpreting and applying the study’s findings. Follow-up qualitative and further quantitative studies may evaluate the long-term evolution of oncologists’ perceptions and attitudes, as well as the impact of AI-driven chatbots on patient outcomes and care efficiency.

### Implications for Practice and Policy Making

Clinicians should be guided by clear, evidence-based protocols and institutional policies that address key concerns such as liability, accuracy, and data privacy. Training both health care providers and patients on the appropriate use of these tools is critical, particularly to ensure that chatbots are leveraged as supportive adjuncts rather than replacements for professional advice. Moreover, a collaborative framework involving regulatory bodies can foster the development of robust validation processes and real-time monitoring systems, ultimately ensuring that chatbot apps enhance, rather than compromise, the integrity of the doctor-patient relationship and patient safety in oncology practice.

### Conclusions

While recognizing the potential of AI-driven chatbots to enhance accessibility of health information and chronic disease management, Chinese oncologists report significant concerns, including liability, misinformation, lack of personalization, privacy and data security risks, and patient readiness. Addressing the challenges requires comprehensive solutions, such as clear policies and guidelines, rigorous testing and validation, institutional endorsement, and robust patient and provider education. Future efforts should focus on resolving the barriers while leveraging the strengths of AI technology to support patient-centered care in a safe, effective, and ethical manner.

## Supplementary material

10.2196/71418 Multimedia Appendix 1 Interview guide.

10.2196/71418 Multimedia Appendix 2 Reflexivity statement.

## Abbreviations

AI: artificial intelligence
LLM: large language model
PI: primary investigator
