# Supplementary material for: Perceptions and Attitudes of Chinese Oncologists Toward Endorsing AI-Driven Chatbots for Health Information Seeking Among Patients with Cancer: Phenomenological Qualitative Study
Source: J Med Internet Res. 2025 Jul 23;27:e71418. doi: 10.2196/71418 (PMC12309621; doi:10.2196/71418)
Supplement: Multimedia Appendix 1 [file jmir-v27-e71418-s001.pdf]

Appendix 1

Interview Guide
Version 3.3

Opening

1. **Express gratitude:**
   "Thank you for taking the time to participate in this interview."
2. **Introduce the study purpose:**
   "This interview aims to understand your perspectives, experiences, and potential concerns regarding the use of AI chatbots based on large language models (LLMs) for patient-driven health information access. Your insights will provide valuable input to better understand the prospects and challenges of integrating this technology into clinical practice."
3. **Confirm privacy protection and recording permission:**
   "This interview will be recorded solely for research analysis purposes. Your identity will remain strictly confidential. May I begin recording?"
4. **Confirm participant's willingness and start the interview.**

Section 1: Background

1. How long have you been working in oncology? What are your primary responsibilities?
2. Are you familiar with AI chatbots developed using LLMs? (These include tools such as ChatGPT and domestic alternatives such as Kimi, iFlyHealth, and Zhiyu Qingyan.) If yes, how did you learn about them?

Section 2: Knowledge and Perceptions

1. What is your understanding of the application of LLM-based chatbots in the field of health information? What potential advantages and limitations do you see?
2. What role do you think these chatbots can play in helping patients access health information?
3. What are your overall views on the accuracy and reliability of this technology?

Section 3: Usage and Recommendations

1. In your clinical practice, have patients ever mentioned using chatbots to access health information? If so, could you share specific examples?
2. Have you ever recommended these tools to patients? If not, why? If yes, under what circumstances would you recommend them?
3. Do you think patients can accurately understand and reasonably use the information provided by these chatbots? Have you encountered cases where patients misunderstood AI-generated advice?

Section 4: Concerns and Barriers

1. Have you had any concerns when recommending or considering recommending these tools? What specific concerns (e.g., accuracy, risks of misuse) do you have?
2. Are you concerned that patients might become overly reliant on these tools?
3. If issues arise after patients act on AI-generated advice, do you think doctors should bear responsibility?
4. Would you feel more comfortable if hospitals or third-party organizations officially introduced and recommended these tools? Why or why not?
5. Have you encountered cases where patients misunderstood or deviated due to using such technologies? If so, could you share an example?

Section 5: Technology Effectiveness and Patient Feedback

1. After patients used AI tools, have you noticed changes in their questioning style, understanding of their condition, or communication habits with doctors?
2. What challenges or problems do you think patients might face when using these tools?

Section 6: Technology Management and Regulations

1. Do you think hospitals or healthcare systems should establish policies or guidelines to regulate doctors' recommendations of AI tools? If yes, what should these guidelines include?
2. Do you believe these AI tools need specialized validation or assessment to ensure they are suitable for patient use?

Section 7: Future Prospects

1. In the future, how do you think these chatbots could better support patients in accessing health information?
2. If these tools become more reliable, would you be willing to recommend them more often? Under what circumstances would you completely trust these tools?
3. What improvements would you such as to see in these AI chatbots to better meet patients' health needs and alleviate doctors' concerns?
4. Do you think it is necessary to educate patients on how to use these tools effectively? If yes, who should be responsible for this education (doctors, hospitals, or AI developers)?

Closing

1. Are there any additional experiences or opinions related to LLM-based chatbots that you would such as to share?
2. Thank you for your time and insights! We will analyze the data and share a summary of the findings with you, if possible.

Notes:

- Adjust the order of questions flexibly based on participants' responses.
- Probe into key points raised by participants, such as:
    - "You mentioned earlier... Could you elaborate on that?"
    - "This differs from other doctors' opinions. Could you share the reasoning behind it?"
- Control the number of follow-up questions based on the specificity of responses and interview duration.

访谈指南

v3.3

开场

1. 感谢参与者抽出时间参与本次访谈。
2. 介绍研究目的：
   “本次访谈旨在了解您对基于大型语言模型（LLM）的 AI 聊天机器人用于患者主导健康信息获取的看法、经验及可能的顾虑。您的意见将为我们提供宝贵的见解，帮助更好地理解这一技术在临床实践中的应用前景和挑战。”
3. 确认隐私保护与录音许可：
   “本次访谈将进行录音，仅用于研究分析，您的身份信息将被严格保密。请问可以开始录音吗？”
4. 确认参与者的意愿并开始访谈。

第一部分：基本背景

1. 您在肿瘤科工作的时间有多长？目前的主要工作职责是什么？
2. 您是否了解基于大型语言模型开发的 AI 聊天机器人？（就是类似 ChatGPT 的 AI 工具，国内有很多类似工具，比如 Kimi、讯飞晓医、智谱清言等）如果了解，您是通过什么渠道得知的？

第二部分：知识与认知

1. 您对基于 LLM 的聊天机器人在医疗健康信息领域的应用有何了解？有哪些潜在的优势和不足？
2. 您认为这些聊天机器人在患者健康信息获取中可以扮演什么角色？
3. 您对这些技术的准确性和可靠性的总体看法是什么？

第三部分：使用与推荐

1. 在您的临床实践中，是否有患者提到过使用聊天机器人获取健康信息？如果有，您能分享一下具体的案例吗？
2. 您是否曾经主动向患者推荐过这些工具？如果没有，是什么原因？如果有，是什么情况下会推荐？
3. 您觉得患者是否能够正确理解并合理使用这些聊天机器人提供的信息？您是否遇到过患者对 AI 建议产生误解的情况？

第四部分：担忧与障碍

1. 您在推荐或考虑推荐这些工具时，是否有过担忧？这些担忧具体包括哪些方面（例如技术的准确性、患者误用的风险等）？
2. 您是否担心患者过度依赖这些工具？
3. 在患者根据 AI 的建议行事后，如果出现问题，您认为医生需要承担责任吗？

4. 如果由医院或第三方机构统一引入这些工具并进行推荐，您是否会觉得责任问题会有所缓解？为什么？
5. 在您的经验中，是否有患者因使用这些技术产生过误解或偏差？如果有，能否分享一个相关案例？

第五部分：技术效果与患者反馈

1. 在患者使用 AI 工具后，您是否观察到患者提问方式、对疾病理解能力或与医生沟通的习惯发生了变化？
2. 您认为患者在使用这些工具时可能会遇到哪些困难或问题？

第六部分：技术管理与规章制度

1. 您认为医院或医疗系统是否应制定一些政策或指引来规范医生推荐 AI 工具的行为？如果是，您认为这些指引应包括哪些内容？
2. 您是否认为需要对这些 AI 工具进行专门的验证或评估，以确保其适用于患者使用？

第七部分：未来展望

1. 您认为在未来，这些聊天机器人可以在哪些方面更好地支持患者的健康信息获取？
2. 如果这些 AI 工具在技术上变得更可靠，您是否愿意更多地向患者推荐？在什么情况下，您会完全信任这些工具的使用？
3. 您希望这些 AI 聊天机器人在未来可以在哪些方面进行改进，以更好地满足患者的健康需求并减轻医生的顾虑？
4. 您是否认为有必要对患者进行教育，以帮助他们更好地使用这些工具？如果是，教育内容应由谁负责（医生、医院、AI 厂商）？

结束

1. 还有哪些与基于 LLM 的聊天机器人相关的经验或看法，您希望补充说明？
2. 感谢您的时间和分享！我们将在分析数据后整理研究结果，并会在可能的情况下与您分享研究总结。

注意事项

- 在访谈过程中可根据参与者的回答灵活调整问题顺序。
- 对参与者提出的关键观点进行追问，例如：
    - “您刚才提到……，能否进一步说明一下？”
    - “这一点与其他医生的看法有所不同，您能谈谈背后的原因吗？”
- 根据回答的具体性和访谈时间控制深度追问的数量。
