# Supplementary material for: Perceptions and Attitudes of Chinese Oncologists Toward Endorsing AI-Driven Chatbots for Health Information Seeking Among Patients with Cancer: Phenomenological Qualitative Study
Source: J Med Internet Res. 2025 Jul 23;27:e71418. doi: 10.2196/71418 (PMC12309621; doi:10.2196/71418)
Supplement: Multimedia Appendix 2 [file jmir-v27-e71418-s002.pdf]

Appendix 2

Reflexivity Statement

As researchers conducting this study, we acknowledge that our professional and personal backgrounds may influence the research process, from data collection to analysis. The primary investigator (PI) is a clinical oncologist with extensive experience in patient care and health education, particularly in oncology. This background has provided valuable insights into the challenges patients face when accessing health information and the potential role of AI-driven tools in addressing these gaps. However, it also introduces a risk of bias, as the PI's prior exposure to AI technologies may shape the interpretation of participants' perspectives.

To mitigate this, we employed several strategies to enhance the rigor and neutrality of the study. The research team included members with diverse expertise, such as qualitative methodology and digital health innovation, ensuring a balanced approach to data interpretation. Regular reflexivity meetings were held to discuss and address potential biases, particularly those stemming from our professional roles or preconceived notions about AI technologies.

During the interviews, we adopted a neutral tone, avoiding leading questions or imposing our own views on participants. We encouraged participants to share their genuine experiences and concerns, even if they differed from our expectations. The use of Colaizzi's method for data analysis further supported a systematic and transparent process, ensuring that findings were grounded in participants' lived experiences rather than the researchers' assumptions.

By acknowledging our positionality and actively addressing potential biases, we aimed to conduct this study with integrity and provide findings that accurately reflect the perspectives of Chinese oncologists regarding AI-driven chatbots in patient health information access.

反思声明

在开展本研究的过程中，我们认识到自身的专业和个人背景可能会对研究过程产生影响，包括数据收集和分析环节。主要研究者（PI）是一名临床肿瘤科医生，具有丰富的患者护理和健康教育经验，特别是在肿瘤领域。这一背景为我们提供了宝贵的洞察，帮助我们更好地理解患者获取健康信息的挑战以及人工智能驱动工具在解决这些问题中的潜力。然而，这也可能导致偏倚的风险，因为 PI 此前对人工智能技术的接触可能会影响对参与者观点的解读。

为了降低这一风险，我们采取了多种策略以增强研究的严谨性和中立性。研究团队包括具备多样化专业知识的成员，例如定性研究方法和数字健康创新，从而确保数据解读的平衡性。我们定期召开反思性会议，讨论并应对潜在偏倚，尤其是源于专业角色或对人工智能技术的预设观念的偏倚。

在访谈中，我们采用了中立的语气，避免使用引导性问题或将我们的观点强加于参与者。我们鼓励参与者分享他们的真实经验和顾虑，即使这些内容与我们的预期不同。采用 Colaizzi 方法进行数据分析进一步支持了系统性和透明化的研究过程，确保研究发现基于参与者的实际经验，而非研究者的假设。

通过认识到我们自身的立场并积极应对潜在偏倚，我们致力于以诚信开展本研究，并提供准确反映中国肿瘤科医生对人工智能聊天机器人在患者健康信息获取中作用的看法的研究结果。
